# Endovascular Repair of Aortic Arch Aneurysm with Surgeon-Modified Fenestrated Stent Graft

**DOI:** 10.1055/s-0039-1677810

**Published:** 2019-02-15

**Authors:** Jesse Manunga

**Affiliations:** 1 Department of Vascular and Endovascular Surgery, Minneapolis Heart Institute at Abbott Northwestern Hospital, Minneapolis, Minnesota

**Keywords:** aortic arch aneurysm, endovascular repair, fenestrated stent graft, surgeon-modified fenestrated graft

## Abstract

The authors describe a technique of treating patients with aortic arch aneurysm using surgeon-modified fenestrated stent graft (SMFSG). The technique is demonstrated in a 80-year-old patient whose aneurysm was successfully excluded with a SMFSG using Cook Alpha thoracic stent graft. The device was deployed, removed from its delivery system, and a fenestration created before being mounted back on the delivery system and constrained. It was transitioned through a series of sheaths before being introduced into its original sheath. The device was implanted via a common femoral artery access site; fenestration cannulated from the left brachial artery and bridged with a stent graft.

## Introduction

Advances in anesthetic and surgical techniques have greatly improved outcomes of patients undergoing open aortic arch reconstruction (OAAR) and made this approach the standard of care. In spite of these advances, the procedure still carries mortality and neurological complication rates as high as 20 and 18%, respectively. 1 2 3 In 1999, Inoue et al reported on the first use of branched endografts for the treatment of arch aneurysm (AA). 4 Since then, various device configurations, including custom-made scallops, fenestrated, and branched endografts, have been evaluated as treatment options for high-risk surgical patients (HRSPs) with AA. 5 6

None of these devices is commercially available in the United States and other parts of the world. There are, however, a few centers investigating arch devices as part of clinical trials. For HRSPs living out of reach of these centers or those who do not meet inclusion criteria into these trials, treatment options are limited. We describe a technique of treating AA with surgeon-modified fenestrated stent graft (SMFSG) using the Cook Alpha proximal thoracic stent graft as a platform. The patient consented for publication of this manuscript.

## Technique

**Fig. 1:**
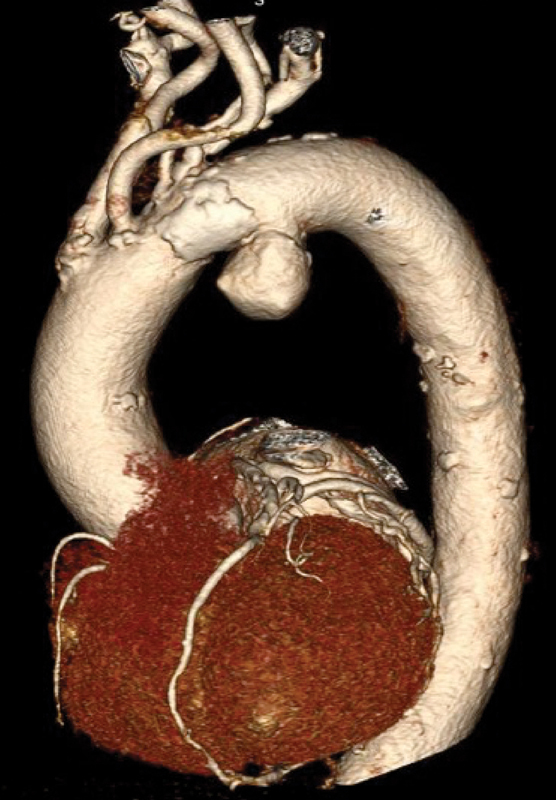
Three-dimensional reconstruction computed tomography angiography of the aortic arch showing a 5.5 cm saccular arch aneurysm. Note the location of the aneurysm—where the ligamentum arteriosum attaches to the aorta. The left vertebral artery (LVA) comes off the aorta just before the left subclavian artery. LVA was not revascularized in this case as the patient was found to have a dominant right vertebral artery and patent basilar artery.

A 80-year-old female presented to her primary care physician with progressive hoarseness. Direct laryngoscopy revealed left vocal cord paralysis. A computed tomography angiography (CTA) revealed a 5.5 cm saccular AA ( Fig. 1 ). Deemed a poor candidate for OAAR by cardiothoracic surgery, she was referred to us for consideration of endovascular repair. Her past medical history was pertinent for tobacco abuse, coronary artery disease, chronic obstructive pulmonary disease, hypertension, hyperlipidemia, and history of infiltrating ductal carcinoma. Careful review of her CTA revealed a few pertinent findings: the left vertebral artery came off the aortic arch, before the left subclavian artery (LSA) takeoff. She had a dominant right vertebral artery ( Fig. 1 ).

After thorough counseling, discussion of risks associated with the procedure, and the lack of long-term data, she was offered repair with a SMFSG using a Cook Alpha thoracic stent graft (Cook Medical).

### Device Modification

**Fig. 2:**
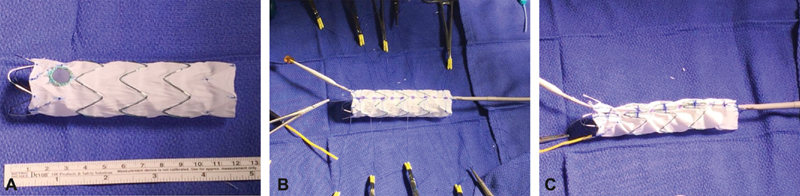
( **A** ) Cook alpha 30 × 109 mm proximal thoracic stent graft deployed and removed from the delivery system. A fenestration created and reinforced with radiopaque snare using 5–0 Ethibond sutures. ( **B** ) The stent graft placed back on the delivery system, one of the 3-nitinol wires withdrawn, rerouted posteriorly through- and -through the fabric using a 20-gauge spinal needle and used as a diameter reducing wire. Note the presence of diameter reducing ties, which are placed as described by Oderich. ( **C** ) Each one of the 3-nitinol wires is used to collapse two uncovered stents. The nitinol wire goes from in inside of the uncovered stent to out, then over to the next and inside out before going back into the hole at the top of the delivery system. Most importantly, note that the fenestration is aligned along *the outer curve* of the precurved inner cannula to assist with alignment of the fenestration with a target vessel.

Before the patient was brought back to the operating room, an Alpha 30 × 109 mm low profile proximal stent graft (LPSG) was deployed on a back table under sterile conditions. The device was removed from its delivery system and an 8 mm fenestration created using an ophthalmologic cautery to accommodate the LSA based on measurements obtained using centerline of flow (TeraRecon). The fenestration was reinforced with a radiopaque snare using a double-armed 5–0 Ethibond locking sutures. The device was then reloaded on its original delivery system and one of the 3-nitinol wires withdrawn from the cannula and used as a diameter-reducing wire ( Fig. 2A–C ). The constraining process was performed as described by Oderich. 7 8

**Fig. 3:**
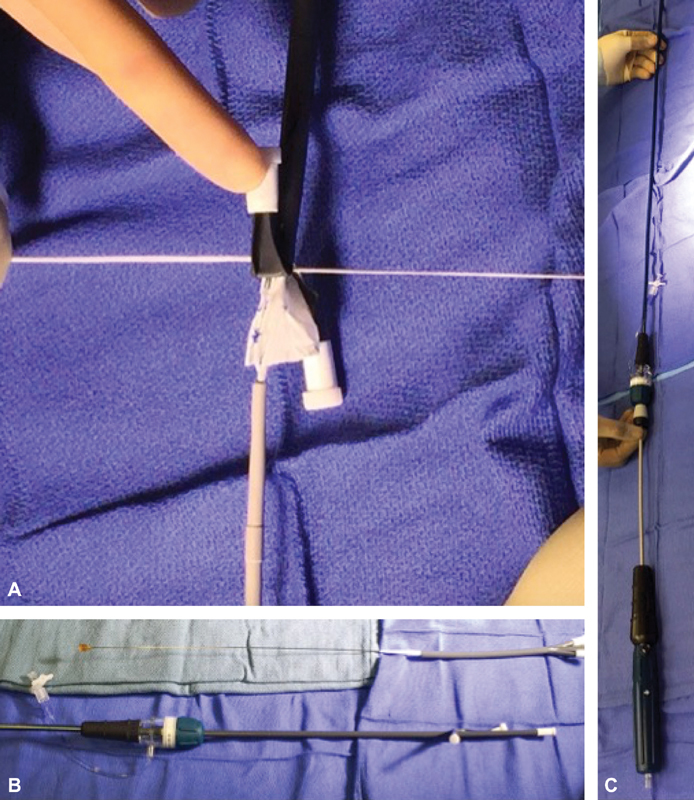
( **A** ) The surgeon-modified fenestrated stent graft and its delivery system are now introduced into a 22 French peel away sheath. ( **B** ) The stent graft is then transitioned to a 20 French peel away sheath. An 18 French peel away sheath is introduced through the valve of the original stent graft sheath. Note that we chose an 18 French peel away sheath for a 16 French original sheath. This allows for the peel away sheath to stay in the valve allowing only the graft to slide in the original delivery system. ( **C** ) The modified graft is now placed back in its original sheath and ready for use.

Unlike the old Cook platform (TX2), the new LPSG comes with 3.5 mm laser cut barbs protruding through the fabric making partial deployment and resheathing impossible without tempering with these barbs and thus compromising the integrity of the device. To overcome this, the modified and constrained LPSG mounted on its original delivery system was introduced into a 22 French peel away sheath then transitioned into a 20 French sheath and subsequently into an 18 French sheath that was advanced through the valves of the original 16 French sheath ( Fig. 3A–C ).

### Implantation

**Fig. 4:**
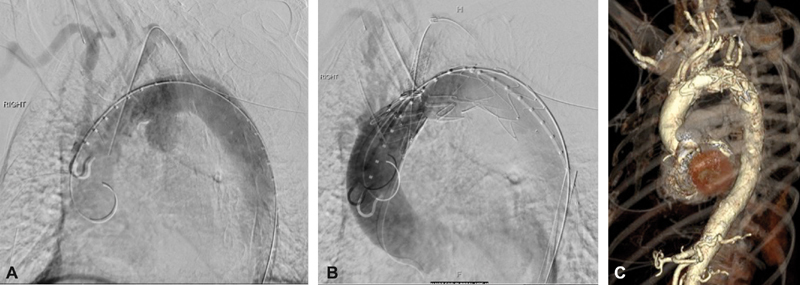
( **A** ) Diagnostic angiography showing the saccular aneurysm and great vessels. ( **B** ) Completion angiography showing exclusion of the aneurysm with excellent perfusion of the target vessel and other great vessels. ( **C** ) Postoperative three-dimensional reconstruction computed tomography angiography showing exclusion of the aneurysm with patent arch vessels.

The operation was performed under general anesthesia. Following percutaneous bilateral common femoral artery (CFA) access and exposure of the left brachial artery (LBA), the patient was systemically heparinized achieving an activated clotting time of > 300 second. Angiography revealed the AA ( Fig. 4A ). The SMFSG was inserted over a Lunderquist wire (Cook Medical) through the right CFA and partially deployed. The diameter-reducing wire allowed for repositioning of the device in various planes to allow alignment and catheterization of the side branch. A slight forward pressure was maintained on the partially deployed fenestrated stent graft from the right groin to avoid device migration, while the fenestration was cannulated using a glide wire (Terumo Medical) and an angled catheter from the LBA access site. A 9 × 38 mm Atrium iCAST stent graft (Maquet) was delivered over a Rosen wire. The constraining wire and delivery system were removed and the SMFSG ballooned. The bridging stent graft was deployed and its proximal end flared with a 10 mm × 2 cm balloon. Angiography ( Fig. 4B ) revealed good perfusion of arch vessels and exclusion of the aneurysm with no endoleak. The CFA was closed percutaneously with Perclose ProGlide devices and the LBA repaired with interrupted 7–0 Prolene sutures. Total fluoroscopy was 417 mGy in 23 minutes and estimated blood loss was < 20 mL.

The patient was neurologically intact upon extubation. She was admitted to the floor and discharged home on postoperative day 2. CTAs (3, 6, 12, and 24 months postoperatively) continue to show a well-positioned device, patent vessels, and a nearly remodeled aneurysm sac ( Fig. 4C ). Her voice is back to baseline.

## Discussion

The quest for minimally invasive approaches to the treatment of AA is driven by a simple fact: an increasing number of these patients present with comorbid conditions rendering them poor candidates for OAAR. While early results of endovascular AA repair with fenestrated/branched endografts are promising, access to these devices remains limited to a handful of centers participating in clinical trials. HRSPs living out of reach of these centers often find themselves with limited treatment options that might include the chimney technique, in-situ fenestration, SMFSG, or expectant management. In our case, a hybrid approach was a viable option, but she refused this approach even after extensive counseling. In some cases, coverage of the LSA has been well tolerated. In this patient, however, LSA revascularization was required to avoid arm ischemia since the left vertebral artery was coming off the aortic arch and thus covered by the stent graft ( Fig. 1 ). Hoarseness was the result of compression of the left recurrent laryngeal nerve by the aneurysm. 9 10 11

The chimney technique carries the advantage of being readily available for use since it utilizes off-the-shelf components. However, the technique has a reported type I endoleak rate of 23%, a chimney graft occlusion rate of 11%, and a significant stroke rate at follow up. 12 13 Proponents of in-situ fenestration advocate its use for aneurysms located on the inner aortic curve to maximize stent graft apposition to the outer aortic wall and minimize the potential for type III endoleak that might result from the use of a short bridging stent graft. 14 However, long-term results of this technique are lacking.

The use of SMFSG for the treatment of complex abdominal and thoracoabdominal aneurysms has been extensively reported. 14 The most commonly used platform for this purpose, Cook TX2, has been discontinued and replaced by the LPSG. The proximal piece of this device comes with protruding barbs that make retrograde resheathing post modification impossible without cutting these barbs—a process that often leads to compromised device integrity and a high incidence of type IA endoleak. The technique described herein allows one to safely modify this device without cutting proximal barbs.

We find it useful to sequentially cannulate one fenestration at the time while deploying the device. This is certainly our approach when using this device to treat thoracoabdominal aortic aneurysm. We always partially deploy enough of the device to allow the cannulation of the first fenestration. This approach affords one the ability to easily rotate and adjust the height of the device in case of fenestration misalignments. Furthermore, it is extremely important to maintain slight forward pressure on the delivery system of the partially deployed SMFSG to avoid migration, while the fenestration is cannulated from the brachial artery access. Only after this maneuver should the entire graft be deployed, the constraining wire removed and the device ballooned. Failure to do so may result in device migration and inability to cannulate the fenestration. In some cases, especially if the device is being deployed in aortic arch zone 0, rapid ventricular pacing may be required to facilitate accurate device landing.

Treatment of arch pathologies using the chimney technique, in-situ stent graft fenestration, custom-made F/B endografts, or SMFSG, is still in its infancy and requires continued technical refinement to achieve ease of use and decrease the rate of procedure-related complications. Patient selection with specific attention to tortuosity of the iliac arteries and aorta as well as the type of aortic arch will prove crucial for a successful and safe implantation of the device. Lastly, these patients require close follow-up to continue evaluating stent graft performance.

## Conclusion

Though feasible, endovascular repair of AA using SMFSG can be a challenging undertaking with potential for significant neurological complications or even death. The procedure should be performed by an experienced team after thorough patient counseling.
